# A novel method for the measurement of glucocorticoids in dermal secretions of amphibians

**DOI:** 10.1093/conphys/coy008

**Published:** 2018-02-16

**Authors:** R M Santymire, M B Manjerovic, A Sacerdote-Velat

**Affiliations:** 1 Lincoln Park Zoo, Conservation & Science Department, 2001 N. Clark St., Chicago, IL 60614, USA; 2 Department of Biology, Virginia Military Institute, 301C Maury-Brooke Hall, Lexington, VA 24450, USA; 3 The Chicago Academy of Sciences, Peggy Notebaert Nature Museum, 2430 North Cannon Drive, Chicago, IL 60614, USA

**Keywords:** ACTH, herpetofauna, stress, trapping stress

## Abstract

Amphibians have been declining in both diversity and abundance due in large part to habitat degradation and the prevalence of emerging diseases. Although stressors can suppress the immune system, affecting an individual’s health and susceptibility to pathogens, established methods for directly collecting stress hormones are not suitable for rapid field use or for use on threatened and endangered species. To overcome these challenges, we are developing an innovative method to collect and measure amphibian glucocorticoid secretions using non-invasive dermal swabs. We tested this methodology using multiple terrestrial, semi-aquatic and fully aquatic species. We swabbed the dorsal side of each animal six times and then induced a stressor of either hand-restraint, ACTH injection, or saline as a control. We then repeated swab collection immediately after the stressor and at 15, 30, 45, 60, 90 and 120 min intervals. Cortisol enzyme immunoassay detected changes in cortisol post-stressor. We also tested this methodology in the field and were successfully able to detect glucocorticoids from multiple species at varying life stages. When using in the field, capture technique should be considered since it may impact stress levels in certain species. Upon further testing, this novel method may be used to greatly increase our understanding of amphibian health especially as disease and environmental changes continue to impact fragile populations.

## Teaser

We are developing a novel method using non-invasive skin swabs to measure stress in several amphibians from terrestrial, semi-aquatic and fully aquatic species both at the zoo and in the wild. Upon further testing, this technique will allow future studies to understand how the environment is impacting amphibian health and susceptibility to disease.

## Introduction

The rapid decline of amphibian populations is attributed to a complex suite of natural and anthropogenic-induced environmental challenges including, but not limited to, predation, resource availability, loss and degradation of habitat, invasive species, climate change and disease outbreaks. These abiotic and biotic factors interact in such a way that interspecific and intraspecific differences may be observed in response to similar environmental threats (Blaustein and Kiesecker, 2002; Hayes *et al.*, 2010). Population level responses also can vary both spatially and temporally such that our understanding of population declines often cannot be attributed to a single factor (Blaustein *et al.*, 2011). For example, the recent emergence and spread of the amphibian fungal pathogen, *Batrachochytrium dendrobatidis* (*Bd*), has led to mass mortalities of native amphibian populations globally (Olson *et al.*, 2013); however, population declines have not been consistent across species, life stage or locations (Fisher *et al.*, 2009; Searle *et al.*, 2011). Therefore, it is important to understand how environmental conditions impact individuals, keying in on factors that may affect disease susceptibility.

It is well known that chronic or repeated stressors can suppress the immune system, affecting an individual’s susceptibility to pathogens. Stress also has been demonstrated to inhibit reproduction, promote severe protein loss (muscle wasting), disrupt secondary cell messengers, cause neuronal cell malfunction and suppress growth (Sapolsky, 1992; Sapolsky *et al.*, 2000; Wingfield, 2005; Wingfield and Romero, 2000; Boonstra, 2005). Psychological and/or physiological stressors result in a cascade of hormonal events that are initiated within the brain activating the hypothalamus–pituitary–adrenal axis (HPA) or HP–interrenal (HPI) axis in amphibians (Rollins-Smith, 2017). First, a perceived stressor results in the hypothalamus releases corticotrophin-releasing hormone, stimulating the anterior pituitary to release adrenocorticotropic-releasing hormone (ACTH) into the bloodstream where it travels to the adrenal/interrenal gland to cause the release of glucocorticoid (GC) hormones, including cortisol and/or corticosterone from the cortex (Reeder and Kramer, 2005). While GC production can be measured to evaluate how individuals are responding to stressors, the challenge is that capturing and restraining animals often induces a stress response and can increase GC production rapidly, in under 5 min for some birds and small mammals (Touma and Palme, 2005; Harper and Austad, 2000; Mostl and Palme, 2002; Millsburgh and Washburn, 2004). In order to reduce this effect, much of the previous wildlife endocrinological research employs non-invasive sampling, such as faecal collection (Monfort, 2003). Not only does this type of sample collection minimally disturb the animal, but faecal samples are a reflection of GCs that occurred over the previous 6–24 h and not from trapping and/or handling, giving an accurate measure of an individual’s response to its environment.

In amphibians, rapid field collection of sufficient volumes of faecal material is unreliable such that current, established methods for directly collecting stress hormones require either a blood draw or urine collection (Kindermann *et al.*, 2012; Narayan *et al*., 2010, 2013; Narayan and Hero, 2011). These methods may be invasive or not well adapted to field studies that require sampling a large number of amphibians across sites. Other methods for measuring amphibian stress require hour-long collection periods, such placing an amphibian into clean water then measuring hormone levels in the water (Gabor *et al.*, 2013) or whole body analysis, which requires euthanasia (Glennemeier and Denver, 2002; Barria *et al.*, 2011), presenting a direct challenge to amphibian conservation projects. Because GCs can be produced in a range of other sites besides the interrenal area, including skin (Zouboulis, 2004), we considered how informative dermal swabs may be for understanding amphibian endocrinology. Therefore, our goal was to develop an innovative and non-invasive method to collect and measure amphibian GC production. Our specific objectives were to: (i) determine if dermal swabs could be used to detect amphibian GC in aquatic, semi-aquatic and terrestrial amphibians; (ii) test if non-invasive dermal swabs could be applicable to field research; and (iii) test ability to detect GCs across multiple life stages of amphibians in the field.

## Materials and methods

### Dermal swab method

Wearing powder-free, latex gloves, we restrained amphibians by, supporting the venter, with the swab collector’s fingers around the sides of the body, making sure not to handle the portion of the mid-back where the skin was swabbed. We immediately swabbed the animal six times dorsally along the mid-back, spanning ~2.54 cm (1 inch) long with a sterile cotton-tipped wood swab (Puritan Cotton-Tipped Applicators, #VWR 10 806-005), which had been pre-cut to ~4 cm. The length of the swabbing was kept consistent when swabbing all amphibians except if the animal was shorter than 2.54 cm; then, it was shortened, but kept consistent for that life-stage depending on the species. We placed swabs in individually labelled 2.0 ml tubes containing 1 ml of 70% ethanol. We then induced an acute stressor consisting of 5 min of manual restraint (in air or in water for fully aquatic species). Manual restraint has been used to induce a mild acute stressor in amphibians for validation of plasma and urinary GC (Coddington and Cree, 1995; Narayan *et al.*, 2010; Narayan, 2013). Following hand-restraint, we immediately collected a second swab in the same manner and location as above, and then repeated the wood swab collection every 15 min for 1 h followed by an additional hour where we collected samples every half hour. This protocol resulted in a total of eight swabs: pre-stress, immediately following stressor (0 min), 15, 30, 45, 60, 90 and 120 min post-stressor. All wood swabs were stored at 5°C until processing.

### Hand-restraint stress test

To determine if GC dermal swab sampling could detect an acute change in cortisol values to hand-restraint, we chose adults (*n* = 14 individuals) of eight species (*n* = 1–2 per species) housed at Lincoln Park Zoo (Chicago, IL), Northern Illinois University (Dekalb, IL), and Chicago Academy of Sciences (Chicago, IL) that represented different habitats and skin types. Species included green treefrogs (*Hyla cinerea*) as a semi-terrestrial/arboreal species, American toads (*Anaxyrus americanus*) as terrestrial with a high density of granular glands, northern leopard frogs (*Lithobates pipiens*) as semi-aquatic, axolotls (*Ambystoma mexicanum*) and a mudpuppy (*Necturus maculosus*) as fully aquatic, and adult red-spotted newts (*Notophthalamus viridescens*) as an aquatic species with a high density of granular glands. Additional hand-restraint test swabs were submitted by collaborators from a captive-housed rough-skinned newt (*Taricha granulosa*), cricket frogs (*Acris crepitans*) and captive-housed larval and juvenile hellbenders (*Cryptobranchus alleganiensis*). All animal experiments conformed to the Guide for Care and Use of Laboratory Animals and were approved by the Lincoln Park Zoo Research Committee (Chicago, IL, USA).

### ACTH challenge

To determine if GC dermal swab sampling could detect acute changes in cortisol value from an ACTH challenge (and saline injection as a control), we used two adult individuals from each of the following species: green treefrogs, American toads and red-spotted newts. In vertebrates, administering ACTH mimics the natural adrenal stress response by causing a rise in cortisol, which returns to baseline within a few hours (Touma and Palme, 2005). Unfortunately, the sex of these individuals was unknown because these species are not sexually dimorphic and not in a breeding situation at the zoo. Using an ACTH challenge to test the utility of GC evaluations has been used in other frog species to validate non-invasive measurements of stress hormones from urine and faeces (Cikanek *et al.*, 2014; Narayan *et al.*, 2010). These authors used a range of 1–5 individuals per treatment of known sex per treatment. Unfortunately, due to housing conditions at the zoo (large exhibits with multiple individuals), we could not collect biological samples, such as blood, urine or faeces.

The swabbing methodology for the ACTH challenge experiment was identical to the protocol outlined above except instead of using a hand-restraint as the stressor, Lincoln Park Zoo veterinarians injected either ACTH or a saline control intraperitoneally (into the coelomic cavity at the junction of the underbelly and thigh, away from the vital organs) using a 25-g needle after swabbing the site with Nolvasan. ACTH was administered at 2.5 ul/g body mass; 0.446 μg ACTH/g bodyweight in 100 μl saline vehicle (0.9% NaCL; Narayan *et al.*, 2010) and saline was administered at 100 μl 0.9% NaCL. Following the injection, we repeated swabbing intervals post-injection for a total of eight swabs.

### Field evaluation

To examine application of this technique for field cortisol sampling, we focused collection efforts on American bullfrogs (*L. catesbeianus*), green frogs (*L. clamitans*) and northern leopard frogs. We opportunistically collected samples from several other species of free-living amphibians including tiger salamanders (*Ambystoma tigrinum*), blue-spotted salamanders (*A. laterale*), spotted salamanders (*A. maculatum*), red-spotted newts, mudpuppies, spring peepers (*Pseudacris crucifer*), boreal chorus frogs (*Pseudacris maculata*), American toads and wood frogs (*L. sylvaticus*). We swabbed both larval and adult *L. clamitans*, *L. catesbeianus*, *L. pipiens, A. tigrinum, A. laterale* and *A. americanus* to confirm that cutaneous GC could be detected across life stages. Pre-metamorphic larvae were gently held in a powder-free gloved hand and swabbed with light pressure across the mid-dorsum, anterior to where the tail fin originates. When working with smaller bodied animals, we adjusted swab length to approximately half an inch (~1.27 cm).

We captured wild amphibians either by Promar collapsible minnow traps set the previous day or via dip-net. Within 3 min of trap checks and wearing fresh gloves for each amphibian, we swabbed the dorsal side of each individual using the same protocol outlined above [i.e. six times dorsally, spanning ~2.54 cm (1 inch) with a pre-cut, sterile cotton-tipped wood swab] and placed the wood swab in an individual 2 ml tube containing 1 ml of 70% ethanol. Typically, once amphibians were in hand from the trap or the dip net, sample collection took <30 s and animals were released at the point of capture. We stored vials at 5°C until processing. To determine if cortisol was detectable in the water, we collected pond water from a subset of our field sites (*n* = 5) throughout northeastern Illinois into 10 ml vials.

### Sample processing and hormonal analysis

We processed all samples at the Davee Centre for Epidemiology and Endocrinology (Lincoln Park Zoo, Chicago, IL). Sample vials containing the wood swab were shaken on a mixer (Glas-col, Terre Haute, IN, USA; setting 60–70 rpm) for 5 min. Then, the wood swab was removed and 500 μl of sample was pipetted into new, pre-labelled 15 × 75 mm^2^ test tube. These aliquots were dried down under forced air in a warm water bath at 60°C. Once dry, 2–3 glass beads were added to each tube, then 500 μl of phosphate buffered saline (PBS; 0.2M NaH_2_PO_4_, 0.2M Na_2_HPO_4_, NaCl) was added. Tubes were vortexed briefly and sonicated for 20 min. Samples were then shaken again on the glas-col mixer (60–70 rpm) for 30 min and stored at 5°C until analysis on an enzyme immunoassay (EIA). To analyse cortisol levels in the water, water samples were ran ‘neat’ on the EIA.

A cortisol EIA was used to measure GCs with the polyclonal antiserum and HRP (R4866; provided by C. Munro, Davis, CA) used at a 1:8 500 and 1:20 000 dilution, respectively. Cross-reactivity to the cortisol antiserum has been previously published (Munro and Stabenfeldt, 1984; Young *et al.*, 2004; Loeding *et al.*, 2011). The cortisol EIA was biochemically validated in the laboratory by demonstrating parallelism using Pearson’s Product Moment correlation to compare the relationship of the parallelism between the cortisol standards and serially diluted swab samples (2 × concentrated to 1:16) for all species separately (Table 1). For the percent recovery, we graphed a scatterplot of the observed over expected values of samples spiked with the cortisol standards and did linear regression to get the equation of the best fit line and *R*^2^ value for each species (Table 1). Assay sensitivity was 3.9 pg/well and intra- and inter-assay coefficients of variation were <15%.

**Table 1:** Biochemical validation for cortisol enzyme immunoassay for all species used for validation and field research

Habitat	Species	Study	Location	Parallelism^a^	Percent recovery^b^
Terrestrial	Green treefrog	Validation; field	Captive; wild	0.996	y = 0.891x + 3.389; *R*^2^ = 0.997; *P* < 0.001
	American toad	Validation; field	Captive; wild	0.988	y = 0.985x + 0.207; *R*^2^ = 0.990; *P* < 0.001
Semi-aquatic	Northern leopard frog	Validation; field	Captive; wild	0.993	y = 0.887x + 0.113; *R*^2^ = 0.999; *P* < 0.001
	Cricket frog	Validation	Wild	0.993	y = 1.180x + 0.798; *R*^2^ = 0.995; *P* < 0.001
Aquatic	Axolotl	Validation	Captive	0.997	y = 0.833x + 6.262; *R*^2^ = 0.993; *P* < 0.001
	Red-spotted newt	Validation	Captive	0.963	y = 0.887x + 0.113; *R*^2^ = 0.999; *P* < 0.001
	Mudpuppy	Validation	Captive	0.992	y = 0.985x + 0.827; *R*^2^ = 0.999; *P* < 0.001
	American Bullfrog	Field	Wild	0.991	y = 1.160x + 2.823; *R*^2^ = 0.999; *P* < 0.001
	Green frog	Field	Wild	0.996	y = 0.891x + 3.389; *R*^2^ = 0.997; *P* < 0.001
	Western chorus frog	Field	Wild	0.985	y = 0.834x + 0.903; *R*^2^ = 0.999; *P* < 0.001
	Spring peeper	Field	Wild	0.995	y = 1.176x + 0.422; *R*^2^ = 0.999; *P* < 0.001
	Tiger salamander	Field	Wild	0.993	y = 1.150x + 0.485; *R*^2^ = 0.998; P < 0.001
	Rough-skinned newt	Validation	Captive	0.996	y = 0.834x + 0.903; *R*^2^ = 0.999; *P* < 0.001
	Blue-spotted salamander	Field	Wild	0.997	y = 1.020x + 1.500; *R*^2^ = 0.999; *P* < 0.001
	Hellbender	Validation	Captive	0.751	y = 1.005x + 0.641; *R*^2^ = 0.999; *P* < 0.001

^a^Parallelism compares the relationship between cortisol standards and serially diluted swab samples (2× concentrated to 1:16) separately for all species calculated using Pearson’s Product Moment correlation.

^b^Percent recovery calculated as a best fit line using a linear regression of observed over expected values when known amounts of cortisol standard is added to a sample.

To ensure the swab was not interfering with the cortisol results, we placed the 4.0 cm wood swab with the cotton tip into 1 ml of 70% ethanol and repeated for a total of 50 swabs (i.e. blank swabs) and kept at room temperature overnight. Then, swabs were processed as above and analysed on the cortisol assay.

Furthermore, because some species of amphibians have been shown to produce skin peptides as a defense mechanism against disease, such as *Bd* (Woodhams *et al.*, 2007), we repeated the percent recovery test with eight known *Bd*+ and eight known *Bd*− frogs to ensure these peptides were not interfering with the EIA results. We found no interference (*Bd*+: y = 0.9908x − 0.9887; *R*^2^ = 0.9997; *P* < 0.001; *Bd*−: y = 0.9911x + 0.8642; *R*^2^ = 0.9996; *P* < 0.001).

### Data analysis

To determine the effect of the acute stressor on adrenocortical activity and the time between HPI axis stimulation and peak cortisol dermal secretions, we determined the absolute change [i.e. the fold increase (Romero, 2004)] by calculating the quotient between the pre-stress samples and the peak samples. For instance, a 1-fold increase above the pre-stress indicates no change in the HPA axis. We considered any time point that was at or above 1.5-fold higher to acute change in cortisol values. To determine if fold increase in cortisol differed between individuals in response to the hand-restraint test, we used *t*-test, if data were normal (using Shapiro–Wilk for normality assumption testing and Levene’s median test for equal variance assumption testing), or a Mann Whitney Rank sum test if data were not normal. Then, for the three species (American toad, red-spotted newt and green treefrog) that had the three stress tests (ACTH, saline and hand-restraint), we compared fold changes in cortisol using an one-way ANOVA for normally distributed data or Kruskal–Wallis ANOVA on ranks if data were not normal (Schell *et al.*, 2013). Correlation (parallelism) and linear regression (percent recovery) for EIA biochemical analysis were performed using Sigma Plot (2008, v 11; Systat Software, Inc., San Jose, CA, USA). Values are presented as mean ± SE. For field stress analysis, we used a Wilcoxon signed-rank test to compare method of capture (e.g. dip-net vs. minnow trap) in adult frogs. All field data analyses were performed in RStudio (v 0.99.486). For all analyses, *P* < 0.05 was considered significant.

## Results

### Laboratory biochemical validation

All species passed the biochemical analysis of parallelism and percent recovery except for the hellbender, which failed the parallelism test as cortisol was unmeasurable even at four times concentration (Table 1). Because of this failure, we repeated the parallelism and percent recovery on our corticosterone EIA (CJM006; Santymire and Armstrong, 2010 for EIA details) and also were not able to measure corticosterone. Therefore, no further sampling and testing was conducted on this species.

For interference testing, we found that the blank 4.0 cm wood swabs with the cotton tip did produce measurable cortisol. Therefore we determined that 1.0 cm long swab produced an average of 53.4 ± 3.1 pg/ml cortisol. Then, we measured all swabs in each sample to the nearest millimetre and subtracted the cortisol/stick value from all samples.

### Hand-restraint test

#### Terrestrial species

**Figure 1:**
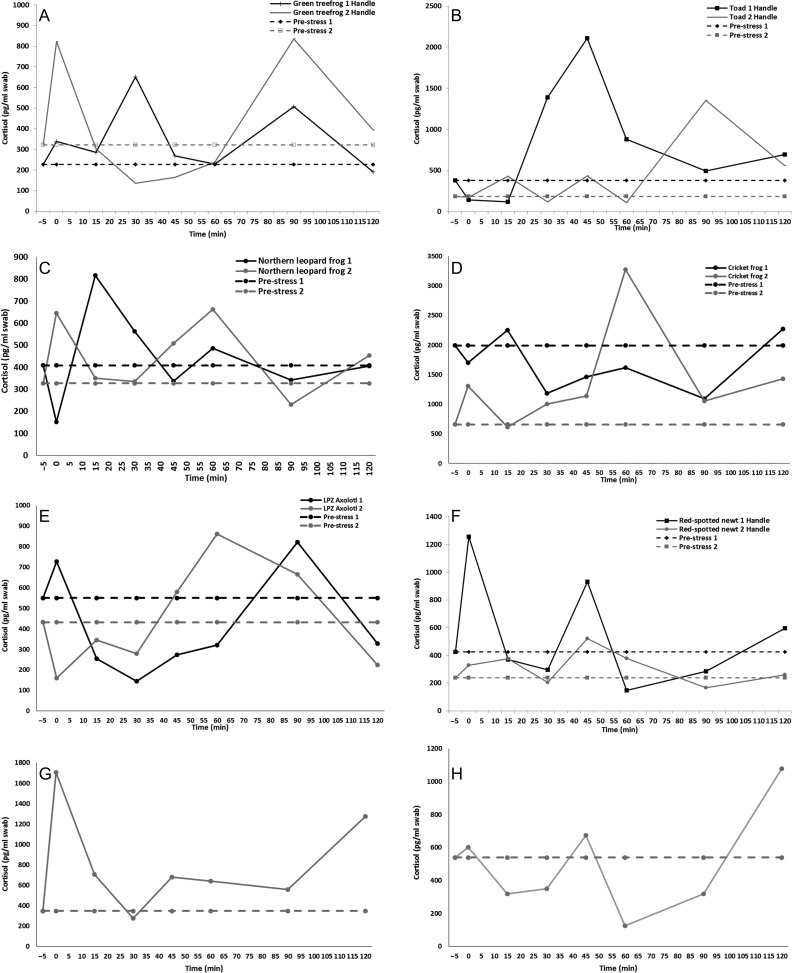
Cortisol (pg/ml swab) response after a 5 min of hand restraint in green treefrogs (**A**), American toads (**B**), Northern leopard frogs (**C**), cricket frogs (**D**), axolotls (**E**), red-spotted newts (**F**), rough-skinned newt (**G**) and mudpuppy (**H**). Pre-stress values (dashed lines) were taken prior to the stressor and 0 min represent sample taken immediately after stressor concluded.

Both the green treefrogs 1 and 2 had similar (*U* = 16.0; *P* = 0.318; range: 0.4–2.9-fold increase) fold increases in cortisol with two peaks ranging from 506 to 834.8 pg/ml occurring 0–90 min post-restraint (Fig. 1A). For American toads 1 and 2, fold increases in cortisol were similar (*U* = 21.0; *P* = 0.710; range: 0.4–7.5-fold increase) and four time points were elevated from 430.0 to 2108.0 pg/ml occurring 15 min through 120 min post-restraint (Fig. 1B).

#### Semi-aquatic species

Both northern leopard frogs 1 and 2 experienced similar fold increases in cortisol (*t*= −1.13; *P* = 0.279; range: 0.4–2.0-fold increase) with at least one peak ranging from 645.4 to 817.2 pg/ml post-restraint that was greater than 1.5-fold higher than pre-stress cortisol (327.3 and 408.6 pg/ml; Fig. 1C). For the cricket frogs, changes in cortisol varied (*U* = 2.0; *P* = 0.002) between the two individuals. Cricket frog 1 had an elevated (3.0-fold higher) pre-stress value (1993.4 pg/ml cortisol) compared to cricket frog 2 (659.2 pg/ml cortisol) and never demonstrated cortisol values above pre-stress value. However, cricket frog 2 had a 2-fold increase post-restraint and had elevated values from 1053.9 to 2275.6 pg/ml cortisol occurring 60 through 120 min post-restraint stress (Fig. 1D).

#### Aquatic species

The two axolotl individuals had a similar (*t*= −0.99; *P* = 0.341; range: 0.3–2.0-fold increase) change in cortisol over time. Specifically, Axolotl 1 never had a cortisol value over 1.5-fold the pre-stress value (549.5 pg/ml cortisol); however, axolotl 2 had one peak >1.5-fold higher than the pre-stress value (431.7 pg/ml cortisol) that occurred 60 min post-restraint stress (861.6 pg/ml cortisol; Fig. 1E). For red-spotted newt 1 and 2, change in cortisol was similar (*t* = −0.84; *P* = 0.934; range: 0.3–3.0-fold increase) and peaks exceeded the pre-stress value (425.0 and 238.3 pg/ml cortisol, respectively) by >1.5-fold occurring at 0 and 45 min and 15, 45 and 60 min post-restraint, respectively (Fig. 1F). The rough-skinned newt demonstrated peaks from the initial pre-stress value (348.8 pg/ml cortisol) at 0 and 15 min and then from 45 through 120 min post-restraint stress (Fig. 1G). The mudpuppy demonstrated one peak (1078.5 pg/ml cortisol) at 120 min post-restraint stress (Fig. 1H). Finally, we were unable to recover measurable cortisol from the hellbender samples even with concentrating the neat samples up to four times.

### Acute ACTH challenge stress test

**Figure 2:**
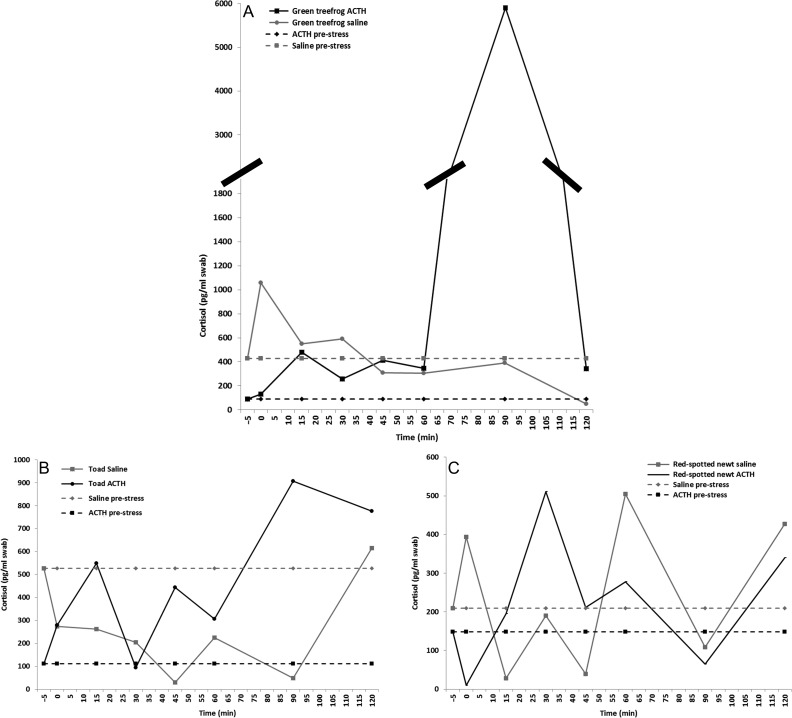
Cortisol (pg/ml swab) response after an ACTH or saline (as a control) injection in green treefrogs (**A**), American toads (**B**) and red-spotted newts (**C**). Pre-stress values (dashed lines) were taken prior to the injection and 0 min represent sample taken immediately afterwards

For the green treefrogs, pre-stress values were 426.7 and 89.0 pg/ml for saline-injected and ACTH-injected individuals, respectively (Fig. 2A). For the saline-injected green treefrog, a 2-fold increase in cutaneous cortisol levels (1056.4 pg/ml) occurred at immediately post-injection. The ACTH-injected green treefrog had elevated cortisol levels (from 2.9- up to 66.4-fold increase) starting 15 min (478.3 pg/ml) through 120 min (341.8 pg/ml) with the peak of 66.4-fold increase (5905.1 pg/ml) at 90 min post-injection. Subsequently, this individual was found dead the following day. Necropsy determined death was not directly attributed to the procedure and with no outwardly signs of poor health, but may have been a complication of a pre-existing condition. The green treefrog sex was determined to be male during the necropsy. When comparing the fold change in cortisol across the ACTH, saline and hand-restraint, the fold-increase post-ACTH (12.64 ± 0.14) was higher (*H*_3_ = 14.02; *P* = 0.003) than the saline (1.09 ± 0.28) and two hand-restraint individuals (green treefrog 1, 1.56 ± 0.28; green treefrog 2, 1.29 ± 0.35).

For the American toads, pre-stress cortisol values for the saline-injected and ACTH-injected individual were 527.0 and 111.4 pg/ml, respectively (Fig. 2B). The saline-injected American toad had no change in cortisol over the entire testing time; however, the pre-stress sample was nearly five times the value of the ACTH-injected individual. And cortisol values did drop from 1.9 to 17.5-fold lower than the pre-stress value between 0 and 90 min. It was back up to pre-stress value at 120 min post-saline injection (Fig. 2B). The ACTH-injected American toad had a 2.5-fold increase (279.9 pg/ml) at 0 min and 4.9-fold increase (549.4 pg/ml) at 15 min. Cortisol values then returned to pre-stress values at 30 min, but became elevated again (2.8–8.1-fold increase) at 45 min and remained elevated through 120 min post-ACTH injection. The change in cortisol was lower (*H*_3_ = 12.99; *P* = 0.005) in the saline treatment (0.45 ± 0.14), than ACTH (4.3 ± 0.98) and two hand-restraint individuals (American toad 1, 2.21 ± 0.72; American toad 2, 2.50 ± 0.90).

For the aquatic red-spotted newt, pre-stress values were 208.9 and 148.1 pg/ml for the saline- and ACTH-injected individuals, respectively (Fig. 2C). The saline-injected individual had a cortisol increase at least 1.9-fold or higher at 0, 60 and 120 min post-injection. The ACTH-injected individual had a 3.5-fold increase at 30 min, 1.9-fold increase at 60 min and 2.3-fold increase at 120 min post-injection. The change in cortisol did not vary (*F*_3,24_ = 0.223; *P* = 0.879) across the three stress tests (1.34 ± 0.17).

### Detection of cutaneous cortisol in wild amphibians

**Figure 3:**
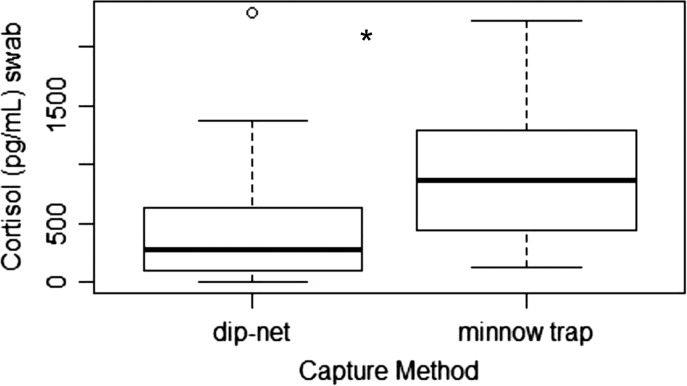
Box plot of cortisol (pg/ml swab) from northern leopard frogs (*Lithobates pipiens*) captured in the field using a dip-net or found in minnow traps set the previous day. Bold line is the median and open circles are suspected outliers. Asterisks indicates a difference (*P* < 0.001) in cortisol concentrations between trapping methods

We successfully detected cortisol from all species collected in the field (Table 2). However, because we collected data from animals that were trapped in minnow traps for an unknown but possibly extended period of time, along with those that were swabbed immediately after being caught in a dip-net, we compared capture type using adults of our targeted species. We collected a total of 138 green frogs (72 dip-net, 66 minnow trap), 131 American bullfrogs (31 dip-net, 100 minnow trap) and 99 northern leopard frogs (44 dip-net, 55 minnow trap). We did not detect a difference in capture methods for green frogs (*H*= 0.84; *P* = 0.36) or American bullfrogs (*H* = 0.87; *P* = 0.35). However, cortisol values from adult northern leopard frogs captured in minnow traps were higher (*H* = 24.02, *P* < 0.001) than samples collected by dip-nets (Fig. 3). We successfully extracted cortisol from species in which metamorphs and larvae were captured (Table 2). No cortisol was detectable in the water samples.

**Table 2:** Mean (±SEM) and range cortisol (pg/ml swab) from amphibian species at various life stages, including adult, metamorphs and larvae, sampled in the field methods via minnow trap

	Adult			Metamorph			Larvae		
Species	*N*	Mean ± SEM	Range	*N*	Mean ± SEM	Range	*N*	Mean ± SEM	Range
American bullfrog	100	600.0 ± 67.4	9.3–5590.3				5	3204.0 ± 2015.9	103.7–11 007.6
American toad	28	890.2 ± 143.5	66.1–2653.1				8	2373.6 ± 491.45	540.0–4605.4
Blue-spotted salamander	34	632.2 ± 80.8	82.2–1720.1				9	1538.12 ± 553.1	626.4–5910.9
Green frog	66	772.9 ± 103.7	8.02–4537.3	2	422.8 ± 190.2	232.6–613.1	13	537.5 ± 105.2	9.3–1180.3
Grey treefrog	2	452.4 ± 273.7	178.72–726.1						
Northern leopard frog	55	908.3 ± 71.6	120.62–2221.3	2	338.9 ± 308.0	31.0–646.9	3	347.8 ± 51.6	249.7–424.9
Red-spotted newt	10	1304.3 ± 349.8	505.7–3757.9						
Spotted salamander	8	927.0 ± 319.9	96.6–2755.6						
Spring peeper	18	1469.3 ± 271.9	115.4–3910.2						
Tiger salamander	27	1047.8 ± 204.0	8.9–3934.38	8	411.8 ± 132.1	26.9–1275.2			
Western chorus frog	21	787.0 ± 104.5	283.3–2378.7						
Wood frog	6	562.4 ± 158.4	176.2–1153.5						

## Discussion

In this study, we have taken the first steps in developing a novel, non-invasive method that uses dermal swabs to detect stress hormones in a variety of amphibians from terrestrial to fully aquatic species. While previous methods utilize collection of biological samples in the form of blood, faeces, whole body, urine and aquatic media (Glennemeier and Denver, 2002; Barria *et al.*, 2011; Kindermann *et al.*, 2012; Narayan *et al.*, 2010, 2013; Narayan and Hero, 2011; Gabor *et al.*, 2013), this is the first time a dermal swab has been successfully used in detecting cutaneous cortisol from captive and wild amphibians. It may be possible to measure hormonal activity on the skin because the skin has been recognized as an endocrine gland (Zouboulis, 2004), both containing hormone receptors and producing hormones (Zouboulis, 2009). However, the change in hormone value may not directly reflect systemic stimulation of the HPI axis, but may be the skin responding to the environment. Additionally, it has been known that thyroid hormones, specifically thyrotrophin-releasing hormone (TRH) and 5-hydroxytryptamin (5-HT), are produced in the granular skin glands of leopard frogs and *Xenopus laevis* (Bennett *et al.*, 1981) and researchers have investigated the role of neuro-endocrine control of skin pigment changes in amphibians (Novales and Davis, 1969). More recently, it has been determined that the catecholamine and epinephrine, regulate rapid pigment change (within 5 min) that occurs in stony creek frogs (*Litoria wilcoxii*) during amplexus (Kindermann *et al.*, 2014).

Here, we used an acute ACTH challenge and a hand-restraint stressor to test the feasibility of this method for monitoring stress physiology in amphibians. These validation methods are activating the HPI axis differently. Specifically, ACTH is a physiological trigger while hand-restraint stress and saline control are biological validations, defined as a challenging procedure, situation or condition (Touma and Palme, 2005), and are individual-dependent (i.e. to what degree is restraint and/or saline injection considered stressful to the individual). Therefore, individuals may have varying responses to the biological stressors. Variability in individual responses to a stressor has been demonstrated in mountain gorilla (*Gorilla beringei beringei*) where the range of elevation in faecal glucocorticoid metabolites went 1.6–9-fold higher than baseline (Eckardt *et al.*, 2016). In captive coyotes (*Canis latrans*), individuals had a range of responses to an ACTH injection (5–30-fold increase), saline injection (2–10 fold increase) and anthropogenic stressors (no change to 18-fold increase; Schell *et al.*, 2013). For this biological stimulation of the HPI axis, we had three individuals (three different species) injected with saline and 14 individuals (eight species) that were given the hand-restraint stress test. We observed varying cortisol levels following the hand-restraint stressor and two of the three individuals/species had at least one elevated point after the saline injection. Three individuals (American toad, axolotl and cricket frog) had no change in cortisol levels which may have resulted from an elevated pre-stress sample. We cannot rule out that the elevated pre-stress samples might have been reflecting increased stress due to transfer from their regular captive housing to holding containers; aquatic species remained in their original tanks. Additionally, unlike using faecal samples for GC analysis from an ACTH challenge, in this study, the animals have to be picked up for each swab to maintain consistent swab pressure during sample collection. This restraint also might induce stress repeatedly and may explain additional cortisol peaks. Overall, 14 of the 17 individuals (eight species not including the hellbender) given a stressor had at least one elevated cortisol value and with a maximum number of six (rough-skinned newt with hand-restraint and green treefrog and American toad post-ACTH) and minimum of one (saline injection: Green treefrog; hand-restraint: axolotl, mudpuppy and northern leopard frog).

When investigating the length of time from the stressor to the increase in cortisol, seven individuals responded immediately post-hand-restraint or saline injection and one had elevated cortisol at 45, 60, 90 and 120 min post-stressor. Previous research that used a similar hand-restraint stressor found an increase in urinary corticosterone in the Fijian ground frog (*Platymantis vitiana*) 2 h post-restraint compared to pre-restraint (Narayan and Hero, 2011). The difference in the timeframe may be attributed to the dermal swabs being a direct reflection of the amount of cortisol circulating in the bloodstream, similar to salivary cortisol, which represents free, unbound cortisol that directly correlates with free plasma cortisol (reviewed in Touma and Palme, 2005; Lane, 2006; Lewis, 2006; Groschl, 2008).

The ACTH injection increased cutaneous cortisol levels to a greater degree than hand restraint or saline alone, particularly in the green treefrog. Similar responses have been observed in other species using different biomaterials. For instance, in harlequin frogs (*Atelopus* spp.), an ACTH challenge resulted in 4–5-fold higher increases in faecal cortisol metabolites 3–4 days post-injection (Cikanek *et al.*, 2014). Using a similar ACTH dose, urinary corticosterone metabolites increased ~4-fold higher (based off of figures) 6 h post-ACTH in Fijian ground frogs, which remained elevated for 2 days (Narayan *et al.*, 2010).

The duration of elevated cortisol also varied by species, individual and type of stressor. Both American toads, in particular, had extended cortisol increases resulting from the hand-restraint. The toad that received ACTH had a similar duration of increased cortisol values but both were significantly higher than the cortisol change in the saline treatment. However, it is difficult to compare to other sample types (i.e. faeces and urine) because sampling only occurs when samples are voided, but dermal swabs may provide similar data as blood. Romero and Reed (2005) investigated the speed in which corticosterone increased in the blood upon capture in six species (five avian and one reptilian; total of 945 individuals) and found that individuals needed to be sampled within 3 min to obtain a value that reflects baseline stress. In some species, a change in cortisol values may be measured on the skin at a similar rate as salivary hormones. In response to ACTH, salivary cortisol has increased by 15 min post-ACTH, in both the chimpanzee (*Pan troglodytes*; Heintz *et al.*, 2011) and ewe (*Ovis aries*; Yates *et al.*, 2010), but that also was the first sample collected post-injection. Both species had elevated salivary cortisol for 3 h, which was not what we found in the three amphibians. ACTH has elicited similar increases in salivary cortisol from 2-fold higher in a squirrel monkey (*Saimiri sciureus*; Tiefenbacher *et al.*, 2003), 5-fold in domestic swine (*Sus scrofa domesticus*; Ruis *et al.*, 2000), 8-fold in chimpanzees (Heintz *et al.*, 2011) to 4-fold in humans (*Homo sapiens*; Contreras *et al.*, 2004).

We also demonstrated that this methodology provides a non-invasive technique to collect cortisol samples in the field with minimal (<5 min) capture time. This method pairs well with other amphibian research, such as collecting swabs for other applications (e.g. *Bd* or ranavirus testing). In addition, the ease and speed of this technique can be applied to sensitive species, such as the Wyoming toad (*Anaxyrus baxteri*) or yellow-legged frogs (*Rana muscosa*), where previous sampling techniques, such as whole body processing (Glennemeier and Denver, 2002; Barria *et al.*, 2011), would preclude data collection. Samples can be collected immediately upon capture greatly improving the speed and ease of data collection compared to previous methods of collecting stress hormones.

Field tests of this technique suggest additional factors may affect results and should be incorporated into analyses. Although not all species exhibited differences resulting from type of capture method (e.g. dip-net vs. minnow trap), northern leopard frogs collected from minnow traps had significantly higher cortisol compared to those that were caught in dip-nets. This likely reflects unknown, potentially long (up to 14 h), durations animals were in the traps. In addition, we detected no cortisol in water samples collected from trapping sites. Other factors such as sex and life stage of animals may influence stress hormone responses and should be considered when developing experimental design and analyses (Hopkins and DuRant, 2011; Narayan and Gramapurohit, 2016). For example, in hellbenders, males are territorial and aggressive during the breeding season and consistently have higher plasma corticosterone compared to females (Hopkins and DuRant, 2011). Hellbenders with skin abnormalities also had higher stress levels compared to animals with normal skin (Hopkins and DuRant, 2011). This is especially important to note as many amphibians that are being swabbed for other diseases may have skin abnormalities that could affect stress hormones.

Of the fifteen species swabbed for either restraint/saline/ACTH stress tests or from the field, hellbenders were the only species where cortisol was not detected. Hopkins and DuRant (2011) examined plasma corticosterone using EIA in adult and juvenile hellbenders and observed peak corticosterone levels were among the lowest reported for amphibians. Similarly with radioimmunoassay (RIA), hellbender corticosterone fell below the detection limit (Hopkins and DuRant, 2011). This illustrates that species must be individually evaluated for a variety of GCs and sampling techniques.

Non-invasive endocrine monitoring in amphibians has vastly improved our understanding of amphibian health and welfare, reproduction, and response to stressors (Narayan and Hero, 2013). In this study, we present a novel and rapid sampling protocol for monitoring stress physiology in several amphibian species. This is the first steps in validating this method but further testing is needed. For example, we observed variation in timing (from 0 to 120 min post-stressor) when cortisol increased on the skin. This could be attributed to differences in species as far as the habitat it lives in (e.g. arboreal vs. fully aquatic) and/or dermal anatomy and physiology. It also could be a reflection of the individual’s experience with human handling. We also observed various cortisol concentration changes from no change (three species; two hand-restraint stress and one saline injection) to a maximum of 66-fold increase. Another consideration was that we only knew the sex of one individual (male green treefrog that died since sex was determined at the necropsy) and it is known that males and females can respond to behavioural and physiological stressors differently (reviewed in Touma and Palme, 2005; Fijian ground frogs, Narayan *et al.*, 2010). Unless species are sexually dimorphic and/or in breeding condition, it may be difficult and/or too invasive to determine sex in the field, but may be important to consider for future studies. Next steps for further validation would be to collect other samples, such as blood, urine or faeces that can support the dermal changes following an ACTH or other acute stressor. Also, because corticosterone is the predominant GC in amphibians (Leboulenger *et al.*, 1986; Kloas and Hanke, 1990), future tests should compare the results of both GCs. Additionally, because ACTH injections have elicited increases in plasma (Jurani *et al.*, 1973) and urine (Narayan *et al.*, 2010) cortisol <1 h and 2 days, respectively, cutaneous sampling should be tested for a longer than 2 h to confirm the lag time between a stressor and increases of cutaneous cortisol. Most importantly, samples should be collected consistently, including how the swab is applied to the animal, to ensure more or less cortisol is not picked up by swab. Additionally, circadian patterns of GCs should be considered when taking samples from diurnal versus nocturnal species since most species have a daily pattern of GC (reviewed in Touma and Palme, 2005). Although we did not detect any ‘background’ cortisol in the water, there is still a possible contamination issue particularly in a closed system like an aquarium; faeces collected in the water may be contaminated and the animal’s skin has been in contact with the water and may be damp. Future studies will have to take this into consideration and ensure pond water on frogs or salamanders is not contaminating the swab. Finally, we suggest using plastic swabs instead of wood to ensure no background levels of cortisol.

Even though this technique needs further validation, it does greatly increase the potential to integrate questions regarding the health and stress of amphibians and improve our understanding of how environmental changes such as noise pollution, contaminants, invasive species and habitat alterations impact amphibian health. It is also recognized that long-term stress can inhibit both reproduction and immunity leading to decreased reproductive fitness and increased susceptibility to diseases. The global threat of diseases such as ranavirus and *Bd* has led to massive field efforts. We suggest that adding a quick stress swab alongside disease swabs will allow researchers to explore additional factors ultimately leading to mortality and population declines. Such a non-invasive and rapid technique for measuring hormones is needed now more than ever for global amphibian conservation. Finally this method has application to more than amphibians and is currently being used to monitor reproductive hormones in zoo-housed pygmy hippopotamuses (*Choeropsis liberiensis*) and is being developed for fish (Santymire pers. communication).
